# Sellar Xanthogranulomatosis in a Two-Year-Old Girl: A Case Report

**DOI:** 10.7759/cureus.49405

**Published:** 2023-11-25

**Authors:** Laith A Ayasa, Sara Rahhal, Ala'a K Najjar, Asad Aldarawish, Izzeddin A Bakri

**Affiliations:** 1 Faculty of Medicine, Al-Quds University, Jerusalem, PSE; 2 School of Medicine, The University of Jordan, Amman, JOR; 3 Department of Neurosurgery, Al-Makassed Islamic Charitable Hospital, Jerusalem, PSE; 4 Department of Pathology, Al-Makassed Islamic Charitable Hospital, Jerusalem, PSE

**Keywords:** touton giant cells, neuromonitoring, frontolateral craniotomy, sellar region, xanthogranuloma

## Abstract

Sellar xanthogranulomas are extremely rare intracranial lesions, particularly in pediatric patients, and their diagnostic and therapeutic challenges prompt thorough investigation. We describe a case of a two-year-old toddler diagnosed with sellar xanthogranuloma, highlighting the challenges encountered in its diagnosis and management. The child presented with symptoms, including headache, ptosis of the left eye, and neurological deficits. Brain computed tomography (CT) and magnetic resonance imaging (MRI) revealed a hypodense sellar lesion. The patient underwent a left pterional craniotomy for resection of the mass. Histopathological examination suggested the diagnosis of sellar xanthogranuloma, characterized by foamy macrophages, giant cells, lymphocytic infiltrates, fibrous proliferation, necrotic detritus, and hemosiderin deposits. Further diagnostic precision was achieved through immunohistochemical staining, including CD1a and langerin, which successfully ruled out the possibility of Langerhans cell histiocytosis (LCH), reinforcing the diagnosis of sellar xanthogranuloma. The successful surgical resection of the lesion led to a favorable outcome, evidenced by the significant alleviation of symptoms as well as the restoration of normal neurological function. Post-operative assessments demonstrated a marked improvement in the patient's quality of life, and there were no observed complications or recurrence of the lesion during the follow-up period.

In summary, our case report not only highlights the rarity and diagnostic challenges of sellar xanthogranulomas but also emphasizes the importance of collaborative medical expertise in achieving accurate diagnosis and successful therapeutic outcomes in pediatric patients. The successful management of this case offers valuable insights into the clinical presentation, diagnostic complexities, and treatment strategy of sellar xanthogranulomas, further enriching our understanding of this uncommon intracranial pathology.

## Introduction

Sellar lesions in the pediatric population pose a significant challenge because of their complex nature and varying clinical manifestations. Among these are sellar xanthogranulomas, which stand out as diagnostically challenging entities owing to the lack of pathognomonic or characteristic imaging features [1]. Additionally, they are considered extremely rare, with a reported prevalence of 0.6% of all sellar lesions [2]. The literature contains a limited number of case reports documenting sellar xanthogranulomas, with even fewer involving pediatric patients. For instance, a review of the literature revealed only a small number of clinical reports confirming sellar xanthogranuloma, and among these, a very limited number were identified in the pediatric population [2,3]. Here, we report a case of sellar xanthogranuloma of the sellar region manifesting as a focal neurological deficit in a two-year-old patient. We have reviewed the literature reported previously to provide insights into the clinical presentation, diagnostic challenges, treatment strategy, and associated postoperative outcomes.

## Case presentation

A two-year-old toddler presented to our hospital for further evaluation and management with complaints of occasional head discomfort, nausea, intermittent vomiting, abdominal pain, and generalized fatigue for two months duration, in addition to eyelid drop in the left eye noticed by her family. Prior to referral, initial management involved antibiotics for suspected acute otitis media, but without any clinical improvement. Further workup included laboratory examinations and a brain computed tomography (CT) scan (performed at an external facility, details unavailable), both of which yielded unremarkable findings. Subsequent imaging at our institution revealed a hypodense sellar lesion. Abdominal ultrasonography revealed enlarged lymphadenopathy.

Over the following weeks, her family noticed an escalation in the severity of eyelid dropping and headaches. Upon seeking medical advice at a pediatric outpatient neurology clinic, the patient was prescribed prednisolone due to suspicion of left abducens nerve palsy, but no clinical improvement was observed.

Upon admission, physical examination revealed an alert and oriented patient with a Glasgow Coma Scale score of 15/15. Notably, the diameter of the left pupil measured 4 mm and was non-reactive, while the diameter of the right pupil was 2 mm and was reactive. Further ophthalmic examination revealed limited abduction of the left eye; it was otherwise unremarkable for refractive errors, squint, fundi defects, optic atrophy, and papilledema. The lenses were clear, and the patient was, otherwise, neurologically intact.

**Figure 1:**
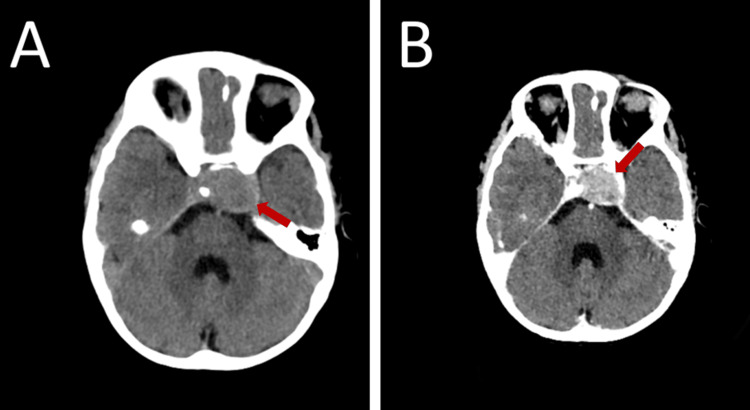
Pre-operative axial pre-contrast CT (A) shows a sellar hypodense lesion (red arrow) extending to the left cavernous sinus with bone changes. After IV contrast injection (B), the lesion (red arrow) shows enhancement. CT = computed tomography, IV = intravenous.

**Figure 2:**
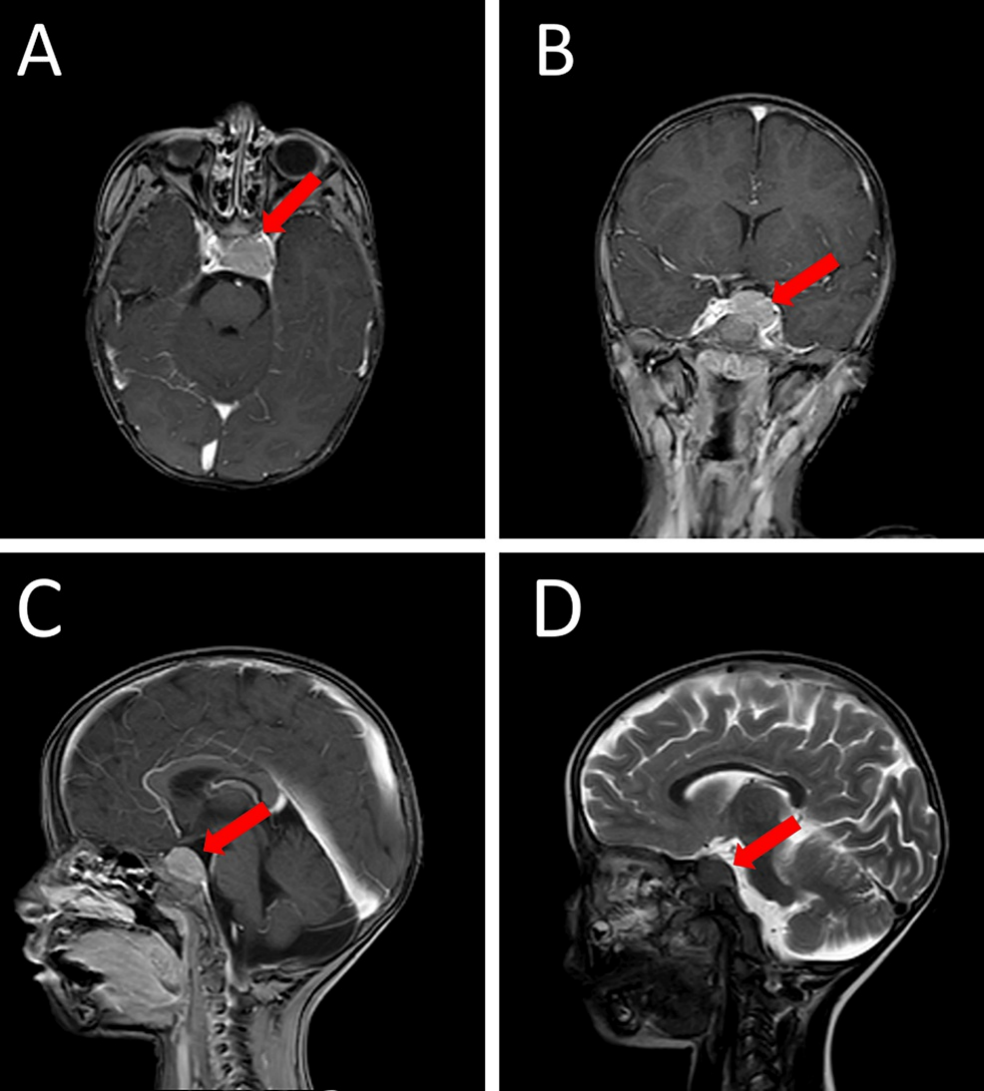
Preoperative MRI of sellar solid lesion (red arrows) extending to the left cavernous sinus, and postoperatively diagnosed as xanthogranuloma of the sellar region. The lesion shows isointense signal in T1WI and hypointense signal in T2WI (D) with homogenous enhancement with gadolinium (A: axial, B: coronal and C: sagittal). The tumor compressed the pituitary gland and the optic chiasma upward. MRI = magnetic resonance imaging, T1WI = T1-weighted images, T2WI = T2-weighted images

Computed tomography (CT) scan revealed a hypodense sellar lesion (Figure 1). Gadolinium-enhanced brain magnetic resonance imaging (MRI) demonstrated a sellar and suprasellar lesion measuring 15 × 24× 13 mm in diameters, and extending to the left cavernous sinus with high signals on T1-weighted images (T1WI) and T2-weighted images (T2WI) (Figure 2). Serum endocrine profile showed normal values of cortisol level, 13.6 nmol/L (normal range: 11.8-80.2 nmol/L), follicle-stimulating hormone, 0.31 IU/L (normal range: 0-5 IU/L), luteinizing hormone, < 0.12 IU/L (normal range: < 0.02 - 0.5 IU/L), adrenocorticotrophin, 36.5 pg/mL (normal range: 9 to 52 pg/mL), growth hormone, 21 ng/mL (normal range: 10 - 50 ng/mL), and antidiuretic hormone, 2.2 pg/mL (normal range: 1.0 - 4.7 pg/mL). An elevated level of prolactin, 32.6 ng/mL (normal range: 3.2-20 ng/mL) was seen. Thyroid profile showed normal thyroid stimulating hormone, 0.83 mU/L (normal range: 0.55-5.31 mU/L), free triiodothyronine 2.15 pg/mL (normal range: 2.0 - 6.0 pg/mL), and free thyroxine, 1.2 ng/dL (normal range: 0.8-2.8 ng/dL).

Given the findings of the MRI, a decision was made to proceed with a left pterional craniotomy for resection of the mass, guided by neuromonitoring and navigation. The transcranial (TC) approach was chosen over the transsphenoidal (TSS) approach due to the superior access allowing for better visualization and resection of the lesion extending to the left cavernous sinus. Multiple samples were obtained and sent for histopathologic examination.

The patient tolerated the surgery well without any intraoperative complications. Neuromonitoring was uneventful with an estimated blood loss of approximately 50 ml. The patient was extubated successfully in the operating room and transferred to the intensive care unit (ICU) for close monitoring. Given the surgical intervention in the sellar region and the patient's prior steroid therapy due to suspicion of left abducens nerve palsy, a hydrocortisone infusion of 50 mg over 24 hours was initiated post-operatively. This was done both as a continuation of the patient's pre-operative steroid regimen and as a prophylactic measure to prevent potential adrenal insufficiency, which can occur post-operatively due to transient disturbance to the hypothalamic-pituitary-adrenal axis

The following day, she was transferred out of the ICU. The patient was started on oral feeding and allowed to ambulate freely. She was kept under observation for five days as per the post-craniotomy protocol. During this period, the patient remained in good general condition with stable vital signs.

**Figure 3:**
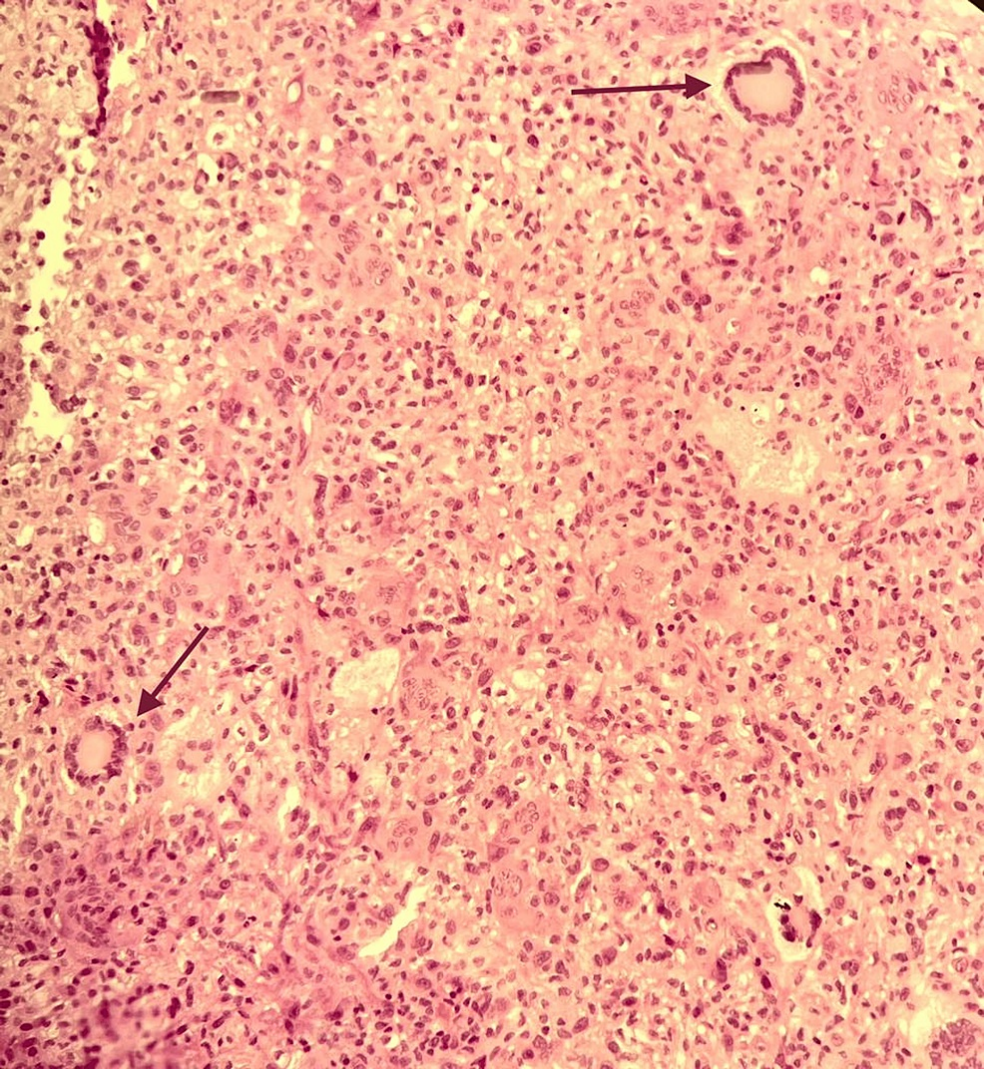
The section shows xanthogranulomatous reaction composed of multinucleated touton giant cells (arrows), aggregates of xanthoma cells, and focal lymphoplasmacytic cell infiltrates. (H&E, original magnification ×20)

**Figure 4:**
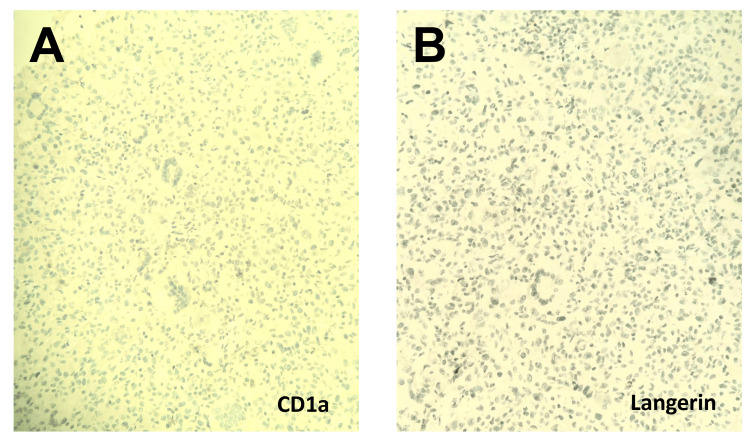
Immunohistochemical staining. (A) CD1a immunostain with adequate control proved to be negative (x 20). (B) Langerin immunostain with adequate control proved to be negative (x 20).

Histopathological examination was compatible with the diagnosis of sellar xanthogranuloma (Figure 3); it revealed a giant cell-rich lesion composed of foamy macrophages, giant cells, lymphocytic infiltrates, fibrous proliferation, necrotic detritus, and hemosiderin deposits. Considering the clinical and radiological findings, the differential diagnoses primarily included sellar xanthogranuloma, Langerhans cell histiocytosis (LCH), Erdheim-Chester disease (ECD), and adamantinomatous craniopharyngioma. To complement the histopathological analysis and definitively rule out differential diagnoses, we conducted immunostaining for CD1a and langerin (Figure 4), which was negative in both cases, further substantiating our diagnosis of sellar xanthogranuloma and effectively ruling out Langerhans cell histiocytosis.

The patient has regular clinical follow-ups at neurosurgery, endocrinology, and ophthalmology clinics with laboratory/radiological investigations. Subsequent follow-ups for the abdominal lymphadenopathy initially observed on sonography revealed a progressive reduction in size, with complete resolution noted at a follow-up visit three months post-operatively. 

## Discussion

The most prevalent tumors in the sellar region among adults are non-functioning pituitary adenomas, whereas craniopharyngiomas are more common among children [4]. Xanthogranulomas, also known as cholesterol granulomas (XG), are inflammatory tumors that rarely manifest in the sellar region; they are more commonly found in the skin and eyes. Sellar xanthogranulomas were first identified in 1988 [5] and later classified as a distinct type of brain tumor by the World Health Organization in 2000 [6].

Xanthogranulomas occurring in the sellar region are exceedingly rare, and due to the lack of comprehensive epidemiological studies, the true incidence remains unknown. For instance, an observational study conducted over 10 years reported that out of 643 patients with sellar or parasellar lesions treated by the Weill Cornell Medical College Department of Neurosurgery in New York, USA, only 0.6% (four cases) were diagnosed with XG of the sellar region [2]. Another study reported that XG accounted for 2.7% (six out of 223) of surgically removed sellar tumors [7].

The existing literature suggests that sellar XG primarily affects adolescents and young adults. In a systematic review performed by Guerrero-Pérez et al., a total of 71 cases of sellar xanthogranuloma were included in the analysis. The average age at diagnosis in these cases was reported to be approximately 35 years old [3]. The present case, involving a two-year-old toddler, stands out due to the rarity of a sellar xanthogranuloma diagnosis at such a young age. Prior reports have documented instances of sellar xanthogranuloma in the pediatric age group, with the youngest cases involving two five-year-old children [8,9]. The median age at diagnosis typically falls around 10 years (range: 5-10) [9,10], further highlighting the unusual nature of our case.

The etiology and pathogenesis of sellar xanthogranulomas are not yet fully understood. Some authors suggest that local tissue damage, such as inflammation, hemorrhage, infarction, or sellar neoplasia, could trigger a reactive degenerative response and granuloma formation [11,12]. Others have proposed that it may be a secondary condition resulting from repeated tissue damage associated with Rathke's cleft cyst or systemic infectious or inflammatory conditions. Xanthogranuloma can occur as an isolated entity or coexist with other sellar lesions such as craniopharyngioma, Rathke's cleft cyst, pituitary adenomas, or hypophysitis [13-15].

Sellar xanthogranuloma poses diagnostic challenges due to its unclear etiology and varied clinical presentation. It can mimic other sellar lesions, making it important to consider this condition in the differential diagnosis. The diagnostic challenges in this case were further compounded by the non-specific nature of the initial symptoms and lack of findings in the initial workup. The patient was initially misdiagnosed with acute otitis media, but showed no clinical improvement. Further investigations were necessary to reach a definitive diagnosis. Another case that highlights the diagnostic challenges of sellar xanthogranuloma involved a patient presenting with intermittent vomiting, occasional headache, and diabetes insipidus. The initial diagnosis was pituitary macroadenoma with hemorrhage in the sellar region. However, histopathological examination after surgical resection confirmed the final diagnosis of sellar xanthogranuloma [16].

Radiologically, sellar xanthogranulomas can be challenging to diagnose pre-operatively due to their clinical and radiological resemblance to more common sellar lesions such as craniopharyngiomas and Rathke's cleft cysts. The defining characteristic radiological findings that can aid in pre-operative diagnosis and operative planning for xanthogranulomas in the sellar region have not been well-described in the literature [1,17]. Therefore, these lesions are often only diagnosed post-operatively. Histologically, xanthogranulomas are characterized by foamy macrophage infiltration, central necrosis, multinucleated giant cells, and hemosiderin deposition [5]. These findings are suggestive of xanthogranuloma, as in our case. 

The clinical presentation of sellar xanthogranulomas can vary, but common symptoms include headache, visual disturbances, and symptoms related to endocrine deficits [3]. Some cases of sellar xanthogranuloma manifest as obstructive hydrocephalus with acute changes in consciousness [18]. Sellar xanthogranulomas are rare in the pediatric population and may be challenging to distinguish from other conditions, such as craniopharyngiomas or Rathke's cleft cysts. In children, the common initial symptoms include headache followed by polyuria, polydipsia, and growth retardation [9,10]. Less frequent symptoms include loss of appetite, blurred vision, and homonymous hemianopsia. Preoperative visual disturbances were noted in multiple cases [9,18].

In the presented case, the patient complained of headache, nausea, intermittent vomiting, abdominal pain, generalized fatigue, and ptosis of the left eye. Notably, we noted an elevated serum prolactin level of 32.6 ng/mL, which is above the expected range for a child of this age. This finding is particularly relevant as it may be associated with pituitary stalk compression or irritation, a common occurrence in sellar masses. Otherwise, no other endocrine abnormalities were observed, as indicated by normal levels of cortisol, follicle-stimulating hormone, luteinizing hormone, adrenocorticotrophin, and thyroid hormones. Sellar xanthogranulomas are typically associated with endocrine deficits. A review by Hernández-Estrada et al. found that the most common presentation in 27 cases was endocrine deficits, often manifested as panhypopituitarism or diabetes insipidus, due to their location and mass effect on the pituitary gland [19]. Additionally, a study conducted by Amano et al. examined the clinical characteristics of seven cases of xanthogranuloma in the sellar region. The study found that six out of the seven cases exhibited endocrine dysfunction, with three of them experiencing an average of four axes in the anterior pituitary affected [13]. In a study conducted by Ved et al., it was found that hyperprolactinemia and hypoadrenalism were the predominant preoperative endocrine deficits observed in patients with pituitary xanthogranulomas. Specifically, these deficits were identified in the majority of patients included in the study (four out of six) [20]. However, there is a documented case where elevated serum prolactin was attributed to a prolactinoma as the cause of sellar xanthogranuloma [21]. This case highlights the variability in endocrine manifestations associated with sellar xanthogranulomas and emphasizes the importance of considering different etiologies in individual cases. Despite these trends, rare cases have been reported where sellar xanthogranulomas present without significant endocrinological abnormalities [8,9,22]. The exact explanation for this discrepancy remains not well understood. One possible explanation could be the size and location of the sellar xanthogranuloma in relation to the pituitary gland. If the lesion is small or located in a specific region that does not directly impinge on the pituitary gland, it may result in minimal or no endocrinological dysfunction. Additionally, the individual variability in the pituitary gland's response to the mass effect of the tumor could also play a role in the absence of endocrinological abnormalities.

Surgical resection is the mainstay of treatment for sellar xanthogranuloma, with gross total resection considered the gold standard for both treatment and diagnosis [20]. Due to the fact that sellar masses are generally smaller at the time of diagnosis, the transsphenoidal approach has been the primary focus in the literature. TSS has the advantages of efficacy, safety, and minimal invasiveness compared with TC surgery. However, the transcranial approach is recommended for suprasellar lesions and when there are contraindications for the transsphenoidal approach [21].

There is limited literature specifically discussing the use of pterional craniotomy for the treatment of sellar xanthogranulomas. In the presented case, the use of a left pterional craniotomy suggests that the surgical team opted for an alternative approach based on the specific characteristics of the patient's sellar xanthogranuloma or other clinical considerations. There is no reported significant difference between the two operating methods regarding outcome or complication rate. However, patients with sellar lesions that are infiltrative in nature treated by transsphenoidal approach were more prone to complications such as CSF fistula [7]. This emphasizes the importance of considering the specific lesion characteristics when choosing the surgical approach, aiming to minimize potential complications and ensure optimal patient outcomes.

In terms of outcomes, improvement in visual symptoms has been reported immediately after effective surgical decompression in some cases [7,23]. However, endocrinological dysfunction is relatively difficult to soon recover to normal; as they may persist postoperatively, or develop [16,20,24,25]. Recurrence or regrowth of sellar xanthogranulomas is rare, as reported in long-term follow-up studies [20,23]. As the long-term prognosis of sellar xanthogranulomas after surgical removal is yet to be evaluated, close clinical and radiological follow-up is indicated.

## Conclusions

Sellar xanthogranuloma, though uncommon, prompts inclusion in the differential diagnosis of lesions involving the sellar and parasellar area, particularly in pediatric patients. Unlike many cases of sellar xanthogranuloma, the patient in our case did not present with significant endocrinological manifestations. This highlights the diverse nature of clinical presentation associated with sellar xanthogranulomas. Additionally, the management of sellar xanthogranuloma in pediatric patients requires a multidisciplinary approach involving neurosurgery, endocrinology, and ophthalmology specialists.
